# Association Between Periprocedural Anticoagulation in Ventricular Tachycardia Ablation and Postprocedural Stroke and Intracranial Hemorrhage

**DOI:** 10.1016/j.jacasi.2025.01.010

**Published:** 2025-03-11

**Authors:** Hisaki Makimoto, Hayato Yamana, Toshiaki Isogai, Hiroki Matsui, Kiyohide Fushimi, Hideo Yasunaga, Takahide Kohro

**Affiliations:** a Data Science Center, Jichi Medical University, Shimotsuke, Japan; b Cardiovascular Center, Jichi Medical University, Shimotsuke, Japan; c Department of Clinical Epidemiology and Health Economics, School of Public Health, The University of Tokyo, Tokyo, Japan; d Department of Health Services Research, Graduate School of Medicine, The University of Tokyo, Tokyo, Japan; e Department of Cardiology, Tokyo Metropolitan Tama Medical Center, Tokyo, Japan; f Department of Health Policy and Informatics, Graduate School of Medical and Dental Sciences, Institute of Science Tokyo, Tokyo, Japan

**Keywords:** complication, intracranial hemorrhage, ischemic stroke, periprocedural anticoagulation, VT ablation

Ventricular tachycardia (VT) ablation is associated with periprocedural risks, including thromboembolic events. Although direct oral anticoagulants (DOACs) have revolutionized stroke prevention in atrial fibrillation (AF) by eliminating the need for routine monitoring, their role in VT ablation remains unclear. Contemporary guidelines have been reticent in prescribing explicit periprocedural and postprocedural anticoagulation paradigms for VT ablation. The STROKE-VT (Safety and Efficacy of Direct Oral Anticoagulant Versus Aspirin for Reduction of Risk of Cerebrovascular Events in Patients Undergoing Ventricular Tachycardia Ablation) study was the first randomized controlled trial to highlight the potential of anticoagulation therapy in reducing cerebral events after VT ablation.^1^ However, because of its limited scale and high incidence of postprocedural thromboembolic complications, there is a pressing need for more extensive research.

## Methods

We used the Japanese Diagnosis Procedure Combination (DPC) database to test the hypothesis that anticoagulation after VT ablation reduces cerebral events within 3 months compared with no anticoagulation. The Institutional Review Board of the University of Tokyo approved the study.

The database encapsulates data of approximately 7 million inpatients annually from approximately 1,100 participating hospitals, representing approximately 50% of all acute inpatient care in Japan.^2^ Using International Classification of Diseases-10th Revision codes, we identified VT patients (I47.0, I47.2) who underwent catheter ablation under a main- or admission-triggering diagnosis of paroxysmal tachycardia between July 2010 and March 2021, excluding patients aged <18 years. The anticoagulation group was defined as patients who received oral anticoagulants between admission and 2 days after the ablation during hospitalization.

The primary endpoints of this study were the incidence of ischemic stroke (transient cerebral ischemic attacks and related syndromes [G45] or cerebral infarctions [I63]) and intracranial hemorrhage (subarachnoid hemorrhage [I60], intracerebral hemorrhage [I61], or other nontraumatic intracranial hemorrhage [I62]) during the initial VT ablation hospitalization or readmissions to the same hospital within 90 days of discharge.

For all statistical assessments, the significance threshold was set at *P <* 0.05. Statistical evaluations were conducted using Stata 18.0 (Stata Corp).

To control for potential confounders, we derived the inverse probability from the propensity scores and implemented weighted analyses (IPW). Propensity scores were estimated using logistic regression regarding age, sex, body mass index (categorized into 3 classes [<18.5, 18.5-25, ≥25 kg/m^2^]), comorbidities upon admission (hypertension, diabetes mellitus, dyslipidemia, heart failure, AF, coronary artery disease, peripheral artery disease, previous history of stroke), antiplatelet medication administration, year of catheter ablation, transseptal puncture, the use of hemodynamic support, and case volume at the institution (4 classes according to the annual number of cases [≤4, 5-9, 10-15, and >15]). To ensure the comparability of groups, we applied the average treatment effect weighting methodology, wherein patients were weighted by the reciprocal of the predicted probability of the treatment they received. A standardized mean difference >10% was interpreted as indicative of an imbalance in patient demographics.

Ischemic stroke and intracranial hemorrhage were compared between the groups. Risk ratios (RRs), along with their 95% CIs, were determined using a weighted, generalized linear model with binomial distribution and log link function. Additionally, the stratified analyses according to antiplatelet drug administration were performed. We compared the outcomes between DOAC and warfarin by an additional IPW within the anticoagulation group. As sensitivity analyses, we performed logistic regression adjusting for covariates instead of IPW.

## Results

We identified 18,885 VT ablation patients, including 12,910 men (68%). The mean age was 59.4 ± 15.5 years. Of these, 4,345 patients (23.0%) received periprocedural anticoagulation (2,316 [12.3%] with warfarin and 2,029 [10.7%] with DOAC), 5,318 (28.2%) were on antiplatelet therapy, and 18,649 (98.8%) received intraprocedural heparin. After the average treatment effect weighting methodology, the patient characteristics were well-balanced between the anticoagulation and nonanticoagulation groups. The standardized difference before and after adjustment were as follows: age (0.635-0.114), proportion of hypertension (0.249-0.043), diabetes mellitus (0.224-0.043), dyslipidemia (0.214-0.028), heart failure (0.505-0.060), coronary artery disease (0.175-0.048), atrial fibrillation (0.541 to −0.011), and stroke history (0.009-0.011).

**Figure 1:**
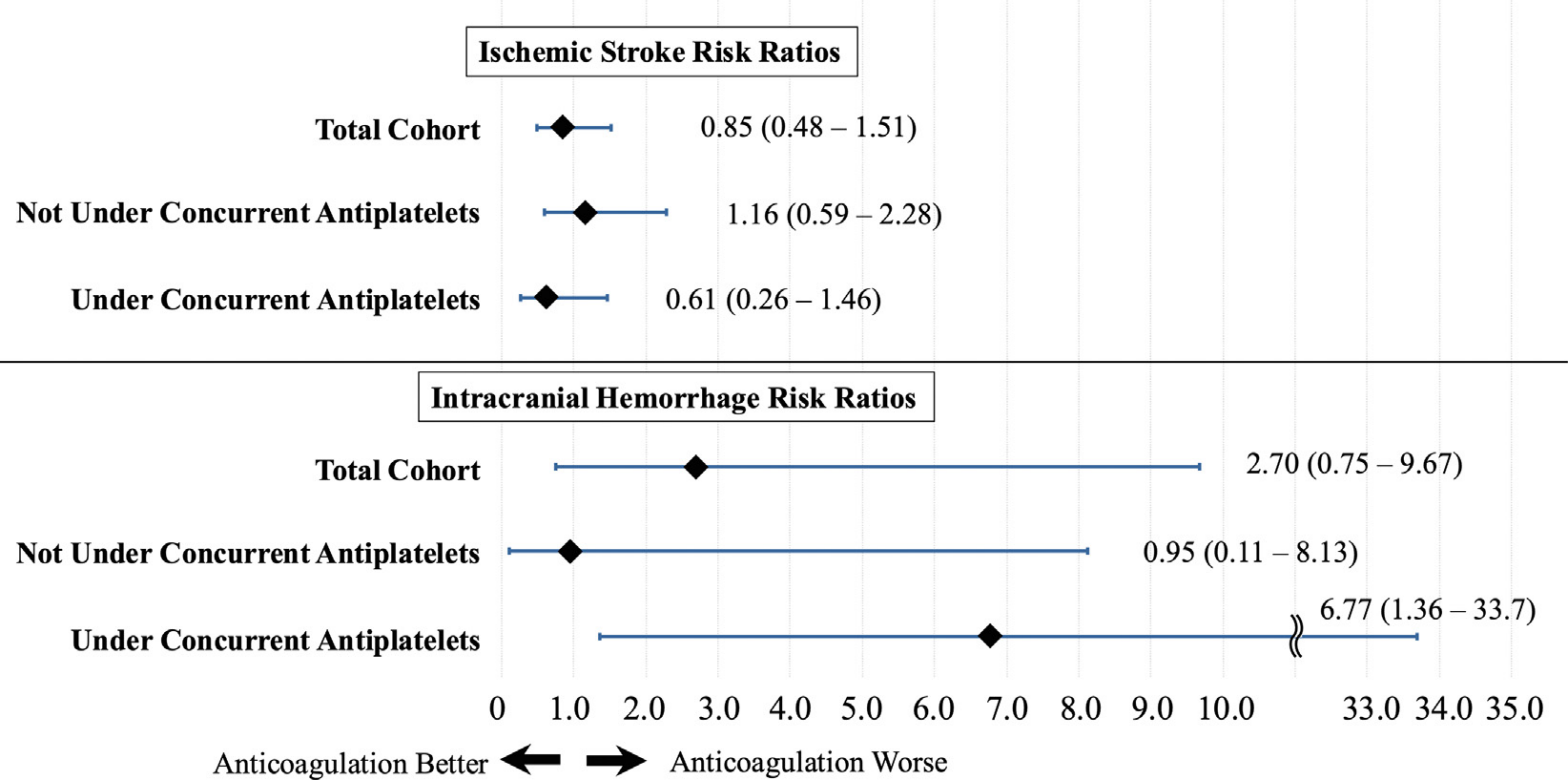
Forrest Plot of Ischemic Stroke and Intracranial Hemorrhage Risk Ratios (Top) The risk ratios for ischemic strokes in the total cohort, subcohorts not under concurrent antiplatelet therapy, and those under concurrent antiplatelet therapy are shown. In all cohorts, periprocedural anticoagulation does not demonstrate any benefits or risk. (Bottom) The risk ratios for intracranial hemorrhage for the total cohort, subcohorts not under concurrent antiplatelet therapy, and those under concurrent antiplatelet therapy are presented. Notably, in the subcohort under concurrent antiplatelet therapy, periprocedural anticoagulation shows an association with an increased risk of intracranial hemorrhage.

Ischemic stroke was observed in 0.44% (95% CI: 0.35%-0.54%) of cases (83 of 18,885). Among these, 22 cases occurred under anticoagulation therapy (0.51% [95% CI: 0.33%-0.77%], 22 of 4,345), while 61 cases occurred without anticoagulation therapy (0.42% [95% CI: 0.33%-0.54%], 61 of 14,540). IPW demonstrated no significant difference in incidence of ischemic stroke between the anticoagulation and nonanticoagulation groups (Figure 1) (RR: 0.85; 95% CI: 0.48-1.51; *P =* 0.59). Intracranial hemorrhage was documented in 15 cases (0.08% [95% CI: 0.05%-0.13%], 15 of 18,885). Among these, 9 cases occurred under anticoagulation therapy (0.21% [95% CI: 0.11%-0.40%], 9 of 4,345), and 6 cases occurred without anticoagulation therapy (0.04% [95% CI: 0.02%-0.09%], 6 of 14,540). IPW showed no significant difference between the anticoagulation and nonanticoagulation groups (RR: 2.70; 95% CI: 0.75-9.67; *P =* 0.13).

Among the 13,567 patients not on antiplatelet therapy, anticoagulation had no significant influence on the incidence of ischemic stroke (Figure 1) (RR: 1.16; 95% CI: 0.59-2.28; *P =* 0.66) or intracranial hemorrhage (RR: 0.95; 95% CI: 0.11-8.13; *P =* 0.97). Among the 5,318 patients on antiplatelet therapy, concurrent anticoagulation did not have a significant impact on the incidence of ischemic stroke (Figure 1) (RR: 0.61; 95% CI: 0.26-1.46; *P =* 0.27). However, incidence of intracranial hemorrhage was higher in those with anticoagulation compared with those without (RR: 6.77; 95% CI: 1.36-33.7; *P =* 0.019).

The occurrences of ischemic stroke (RR: 0.89; 95% CI: 0.37-2.18: *P =* 0.81) and intracranial hemorrhage (RR: 0.47; 95% CI: 0.11-1.96: *P =* 0.30) did not differ between the patients under DOAC and under warfarin.

The sensitivity analyses yielded similar results for ischemic stroke (RR: 0.83; 95% CI: 0.47-1.44; *P =* 0.50) and intracranial hemorrhage (RR: 2.50; 95% CI: 0.75-8.36; *P =* 0.14).

## Discussion

The interpretation of these results requires consideration of the limitations inherent to the retrospective observational studies using pre-existing databases. Procedural details, such as interrupted or uninterrupted anticoagulation, duration, radiofrequency time, radiofrequency settings, induction outcomes, approach and radiofrequency sites in the ventricle (left or right), extent of low-voltage areas, and ablation endpoints, were unavailable. There were also multiple unmeasured clinical covariates such as left ventricular function and dimensions. Additionally, although periprocedural anticoagulant use was examined, exact durations could not be assessed.

In our study, the incidence of ischemic stroke was 0.44% (83 of 18,885), consistent with previously reported incidences of 0.5% to 1.0% after catheter ablation, including VT ablation.^3^ Furthermore, a recent report on the use of antiplatelet therapy after VT ablation showed a very low incidence of 0.3%, which also aligns with our findings.^4^

Our findings contrast with those of the STROKE-VT trial. The randomized controlled study exclusively used DOACs, and its highly specialized centers may have enrolled a specific patient cohort at a high-risk of embolic events. Further research is necessary for decision-making in periprocedural anticoagulation of VT ablation.

We observed very low incidence of periprocedural intracranial hemorrhage at 0.08% (15 of 18,885). A review of 50 studies analyzing complications after VT ablation reported that major bleeding, including intracranial hemorrhage, occurred at a rate of 0.06% to 0.13%, with which our data closely align.^5^ Our study may have been underpowered to assess the effects of anticoagulants on this outcome. Additionally, a wide CI indicated instability for our subgroup analysis, which suggested the increased risk of intracranial hemorrhage in patients concurrently taking antiplatelet drugs.

In conclusion, this observational study suggested that periprocedural anticoagulation therapy for VT ablation was not associated with stroke. Further large-scale studies are required to assess the periprocedural anticoagulation strategy for VT ablation.

## Funding Support and Author Disclosures

This work was supported by grants from the Ministry of Health, Labour and Welfare, Japan (23AA2003 and 22AA2003), and the Cross-ministerial Strategic Innovation Promotion Program (SIP) on “Integrated Health Care System” (JPJ012425). HiMa has received speaker honoraria from Daiichi-Sankyo Co, Ltd. All other authors have reported that they have no relationships relevant to the contents of this paper to disclose.
